# Case report: exome sequencing identified mutations in the *LRP5* and *LGR4* genes in a case of osteoporosis with recurrent fractures and extraskeletal manifestations

**DOI:** 10.3389/fendo.2024.1475446

**Published:** 2024-10-25

**Authors:** Poonam Mehta, Aakriti Sharma, Anupam Goswami, Sushil Kumar Gupta, Vaibhav Singhal, Kinshuk Raj Srivastava, Naibedya Chattopadhyay, Rajender Singh

**Affiliations:** ^1^ Division of Endocrinology, The Council of Scientific and Industrial Research (CSIR)-Central Drug Research Institute, Lucknow, India; ^2^ Academy of Scientific and Innovative Research (AcSIR), The Council of Scientific and Industrial Research (CSIR)-Central Drug Research Institute, Lucknow, India; ^3^ Department of Endocrinology, Sanjay Gandhi Post Graduate Institute of Medical Sciences, Lucknow, India

**Keywords:** osteoporosis, low BMD, recurrent fractures, LRP5, LGR4

## Abstract

**Background:**

Genetic mutations have been reported in a number of bone disorders with or without extra-skeletal manifestations. The purpose of the present study was to investigate the genetic cause in a middle-aged woman with osteoporosis, recurrent fractures and extraskeletal manifestations.

**Methods:**

A 56-year-old Indian woman presented to the clinic with complaints of difficulty in walking, recurrent fractures, limb bending, progressive skeletal deformities, and poor overall health. At the age of 37, she had experienced severe anemia with diarrhea, significant weight loss, knuckle pigmentation, and a significant loss of scalp hair. She had received multiple blood transfusions and parenteral iron supplementation with normalization of hemoglobin. Subsequently, she had premature menopause at the age of 37. She died at the age of 61 due to liver failure. Exome sequencing followed by Sanger sequencing were undertaken to identify the potential pathogenic mutations.

**Results:**

Genetic investigation identified likely pathogenic mutations in the *LRP5* and *LGR4* genes. Out of the two mutations, the heterozygous mutation (c.1199C>T) in the *LRP5* gene resulted in a non-synonymous substitution of alanine with valine at the 400^th^ position, and the second mutation (c.1403A>C) in the *LGR4* gene led to a non-synonymous substitution of tyrosine with serine at the 468^th^ residue of the protein. The minor allele frequencies of the c.1199C>T (LRP5) substitution in the 1000 genomes and IndiGenomes databases are 0.0003 and 0.001, while the c.1403A>C (LGR4) substitution has not been reported in these databases. Various in silico prediction tools suggested LGR4 mutation to be pathogenic and LRP5 mutation to be likely pathogenic.

**Conclusion:**

Heterozygous mutations in the *LRP5* and *LGR4* genes had additive deteriorative effects on BMD, resulting in recurrent fractures and bone deformities, and extended the effect to extraskeletal sites, contributing to the poor overall health in this patient.

## 1 Introduction

Osteoporosis is a major public health issue, with significant morbidity and mortality worldwide. Osteoporosis is characterized by low bone mass and deterioration of bone microarchitecture with increased skeletal fragility (1). Primary osteoporosis may stem from defective collagen formation, bone mineralization, and osteoblast differentiation and function (2), resulting in poor bone formation, low bone mineral density (BMD), altered bone turnover, impaired bone microarchitecture, and an increased risk of fracture (3). Both osteoporosis and osteogenesis imperfecta (OI) exhibit brittle bones as their primary characteristic features. However, while osteoporosis primarily affects bone density and strength, OI cases may present with additional extra-skeletal manifestations such as dentinogenesis imperfecta, blue sclera, and hearing loss (4). The typical OI is characterized by recurrent fractures without significant trauma in childhood, while osteoporosis without the typical characteristics of the OI may present with low BMD and recurrent fractures in the juvenile or adult stage.

BMD is a highly heritable polygenic trait, with up to 80% of variations in BMD explained by genetic variations (5). GWAS studies have identified 144 genes that influence bone quality, bone turnover, and the risk of osteoporosis (3). Genetic polymorphisms in more than 80 genes have been identified to affect BMD in a quantitative manner (6, 7). While the risk of low BMD and fractures is governed by several genes in a polygenic manner, mutations in certain master regulators may significantly increase the risk of osteoporosis, making them primary contributors to primary osteoporosis (6). Technical advancements in genetic analysis, particularly the introduction of whole exome sequencing, have accelerated research into understanding the genetics of primary osteoporosis. These advancements have enabled the identification of mutations in several genes that lead to osteoporosis, distinct from the characteristic features of OI, thus providing differential diagnosis between the two pathologies (8, 9). India has a high prevalence of vitamin D deficiency due to multiple reasons: dark skin, poor exposure to sunshine due to cultural reasons, poor dietary calcium intake, lack of food fortification, etc. Vitamin D deficiency-induced osteomalacia is not uncommon in India, and it exacerbates the high prevalence of osteoporosis in postmenopausal women.

Despite significant advances in the genetics of osteoporosis, the genetic causes in more than 50% of the cases with low BMD and osteoporosis remain unknown. Since mutations in the coding regions of the genome explain up to 85% of human diseases (10), we hypothesized that exome sequencing has the capability to identify causative genetic causes in idiopathic osteoporosis cases. In the present study, we used exome sequencing to investigate a case with recurrent multiple vertebral and appendicular fractures with no typical features of OI, severe iron deficiency anemia, and acute liver failure. The study identified heterozygous mutations in the *LRP5* and *LGR4* genes, which could explain the phenotype in the case under investigation.

## 2 Materials and methods

### 2.1 Case description

A 56-year-old Indian woman presented to the Bone Health Clinic, Department of Endocrinology, Sanjay Gandhi Post Graduate Institute of Medical Sciences (SGPGIMS), Lucknow, India, with complaints of difficulty in walking, recurrent fractures, and limb deformities.

At the age of 27, she suffered a fracture in the left humerus (shaft) after falling from a motorbike. She underwent open reduction and internal fixation with metal rod implantation after the failure of conservative management. However, she developed a malunion of the left humerus.

At the age of 37, she experienced severe anemia (Hb-2.4 mg/dl) with diarrhea, significant weight loss (about 20 kg over 6 months), knuckle pigmentation, and significant loss of scalp hair. She was diagnosed with a severe iron deficiency. There was no history of bleeding diathesis, abdominal pain, jaundice, steatorrhea, fever, cough, skin rash, or azotemic symptoms. She received multiple blood transfusions and parenteral iron supplementation with normalization of hemoglobin. Subsequently, she had premature menopause at the age of 37. At 44 years of age, she reported experiencing cold sensations in both feet, progressive difficulty in walking and standing, knee buckling, and bilateral pedal oedema. She also noticed progressive bending of both legs. Further evaluation revealed bilateral tibia and fibular fractures (without antecedent history of trauma) with permanent deformities of the tibia and fibula. She denied having ever experienced kidney stones, neuropsychiatric symptoms, stomach pain, eye problems, skin rash, polydipsia, or cushingoid characteristics. In addition, she denied having a history of tuberculosis, thyroid dysfunction, diabetes mellitus, hypertension, arteriosclerotic cardiovascular disease, chronic renal or hepatic disease, and substance abuse. Her family history was unremarkable; no member had been documented to have suffered from fractures, kidney stones, or deformities. Evaluation revealed osteoporosis, normal total serum calcium, inorganic phosphorus with markedly elevated alkaline phosphatase (599IU/l, reference range 50-150 IU/l) and intact parathyroid hormone (257 pg/ml; reference range 16-65pg/ml). ^99m^Tc-MDP whole body bone scan suggested a metabolic superscan suggestive of osteomalacia. She received elemental calcium (1000 mg daily) and cholecalciferol (300,000 IU intramuscularly single dose), and thereafter, she improved.

In 2019, at the age of 57 years, she was subjected to a metabolic workup. Physical examination revealed a conscious and oriented lady with normal vital parameters (pulse rate: 93/Min, blood pressure: 144/65mmHg, respiratory rate: 14/min). She had multiple bony deformities; kyphoscoliosis with crowding of the ribs, shortening of the left upper limb with humerus deformity, bilateral anterior tibial and fibular bowing, the left genu valgum, and the right genu varus deformity. She also had restrictions of movement on both shoulders with difficulty raising her arms above shoulder level. The skin below the mid-shaft regions of the tibia was hyperpigmented, shiny, non-tender, oedematous, and showed loss of hair. She also had enamel hypoplasia. Cardiac, respiratory, and abdominal examinations were unremarkable. She had proximal muscle weakness in both lower limbs with normal sensory system examination findings. Serum calcium, inorganic phosphorus, alkaline phosphatase, albumin, and creatine were normal. Serum 25OHD and iPTH were 116 nmol/l and 4.07pmol/l, respectively. She had primary auto-immune hypothyroidism [serum TSH- 27 μIU/ml; (reference range 0.4-4.5 μIU/ml), anti-thyroid peroxidase antibody (anti-TPO antibody) 100 IU/l; (reference range <35 IU/l)], with no biochemical or histopathological evidence of coeliac disease or distal renal tubular acidosis (ammonium chloride challenge test). Serum follicle stimulating hormone was 76 IU/l, suggesting ovarian failure, which was compatible with her age and the fact that she had menopause at the age of 37 years. Dynamic bone histomorphometry could not be done due to its non-availability. She was treated with levothyroxine, calcium, and cholecalciferol.

She was re-evaluated at the age of 60. She has normal serum total calcium, inorganic phosphorus, alkaline phosphatase, albumin, and creatinine. Serum iPTH and 25OHD were 4.42 pmol/l and 72.19 nmol/l, respectively. Serum TSH was 9.12 μIU/ml after a replacement dosage of levothyroxine 100 μg daily. BMD measurement (DXA, Hologic Discovery) showed T scores at the lumbar spine (L1-L4), total hip and femoral neck of -1.3, -2.0, and -1.1, respectively. IVA showed a mild wedge fracture in dorsal vertebrae D7 and D9. Skeletal survey of the whole body showed focal calvarial thickening in the left parietal region, decreased pelvic inlet calibre with a V shaped inlet, bilateral reduced hip joint space, focal anterior bowing of the tibia and fibula diaphysis near the knee end, dorsal spondylosis, and a left humerus malunited fracture with deformity and shortening. She was diagnosed with idiopathic osteoporosis with primary hypothyroidism and recovered from vitamin D deficiency induced osteomalacia.

Six months later, she was admitted to another hospital with recurrent vomiting and jaundice and was found to have acute liver failure. She was conservatively managed for acute liver failure. After suffering long complicated illness for several years, she died at the age of 61 due to acute liver failure.

### 2.2 Exome sequencing and analysis

The study was approved by the Institutional Human Ethics Committee of the Central Drug Research Institute, Lucknow (CDRI/IEC/2020/A3). Informed written consent of the patient for participation in this study was obtained. All experiments on this patient were performed in accordance with the Declaration of Helsinki (1964) for the medical community. Whole exome sequencing was performed on the patient’s blood sample. Genomic DNA was isolated from a blood sample using the MasterPure Complete DNA and RNA Purification Kit (Epicentre). The exonic regions were captured using the SureSelect Human All Exon V6 kit (Agilent Technologies) and sequenced on Illumina NovaSeq (Illumina, San Diego, CA, USA) using 2*150 bp paired end chemistry. To perform the exome data analysis, standard protocols and software were utilized. For the alignment of reads, Burrow Wheeler Aligner (http://bio-bwa.sourceforge.net/) and Samtools (http://samtools.sourceforge.net/) were used. The sequenced reads were aligned to the human reference genome (hg19) downloaded from the UCSC genome browser (http://genome.ucsc.edu/). The variant calling was performed using the GATK variant caller and to annotate the VCF file, wANNOVAR (https://wannovar.wglab.org/) was used. For filtering the potential candidate variants, the minor allele frequency of <0.001 was applied. The in-silico scores like SIFT and PolyPhen2 were set to “damaging” and “possibly damaging” predictions. All synonymous variants were filtered out. Shortlisted variants were further scrutinized for genes previously reported in low BMD, osteoporosis, and OI phenotypes, followed by the scrutiny of those in close association with the master bone regulator genes and those having close connections with bone turnover and metabolism.

### 2.3 Sanger sequencing

For the confirmation of potential pathogenic mutations, we conducted sanger sequencing. The primer design was authenticated by NCBI’s primer design tool, PrimerBlast (https://www.ncbi.nlm.nih.gov/tools/primer-blast/). The reference sequences for the *LRP5* and *LGR4* genes were obtained using the Ensembl genome browser (http://www.ensembl.org/). The mutation site, c.1199C>T, in the *LRP5* gene was amplified using forward primer - ATGAACGAGTGACCATGTAGCAT and reverse primer - AAGACAGTGTCCCAGAATGACAG, and the mutation site, c.1403A>C, in the *LGR4* gene was amplified with the forward and reverse primers, viz., 5’GAGAAAGTCTCGTTGTCATCTGATACC3’ and 5’AACTCTTAGCCTCAAATGACCCTC3’. To amplify the target sites, we used AmpliTaq Gold 360 master mix using the Veriti PCR system (Applied Biosystems, Foster City, CA). We purified the PCR products using the QIAquick PCR Purification kit (QIAGEN, Hilden, Germany), and Sanger sequencing was performed using BigDye chain terminator cycle sequencing chemistry.

### 2.4 In silico mutation analysis

American College of Medical Genetics (ACMG) criteria was applied for interpretation of the observed mutations. We used Varsome (https://varsome.com/) to test the observed mutations on various in silico pathogenicity scales. To assess the potential pathogenicity of the mutation, we employed AlphaMissense (11) and MetaDome (12) prediction tools. For structural modelling of the protein and its complexes, we utilized AlphaFold 3 (13), followed by visualization and advanced analysis using UCSF ChimeraX (version 1.7.1). The modelled structures were employed to predict the changes in free energy (ΔΔG) due to the mutation using DDMUT (14) which provides an estimate of the ΔΔG values linked to the mutation. Additionally, we predicted the impact of the mutation on protein-protein interactions through DDmut-PPI and ScanNet to further elucidate the potential functional consequences of the mutation. To assess the effect of mutation on protein stability with a closer look at intramolecular interactions, we used the DynaMut2 tool. For DynaMut2 prediction (15), we used AlphaFold structure for LRP5 available at https://alphafold.ebi.ac.uk/download, and crystal structure PDB file for LGR4 available at https://www.rcsb.org/structure/4KT1.

## 3 Results

**Figure 1:**
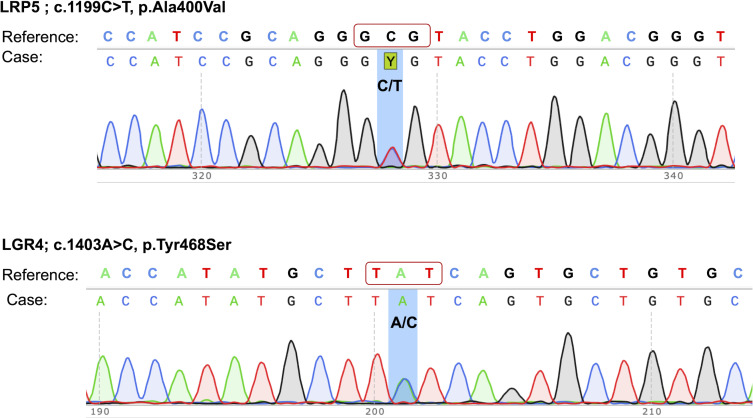
Sanger sequencing electropherograms of pathogenic mutations in the *LRP5* and *LGR4* genes. The mutations are highlighted by a background shade.

Our study aimed at identifying the causative mutation(s) for bone fragility and health deterioration in this woman. Out of the two pathogenic mutations identified in this case, the heterozygous mutation (c.1199C>T) in the *LRP5* gene resulted in a non-synonymous substitution of alanine to valine at the 400^th^ position, and the second mutation c.1403A>C in the *LGR4* gene led to a non-synonymous substitution of tyrosine with serine at the 468^th^ residue of the protein. Both of these mutations were confirmed by Sanger sequencing of PCR amplicons in forward and reverse directions (**Figure 1**).

Variant annotation data for both of these mutations are presented in **Table 1**. The LRP5 p.Ala400Val mutation has been assigned an rsID of rs201320326 and has been reported in homozygous form in a case of familial exudative vitreoretinopathy (16). This mutation has very low frequencies in South Asian and Indian populations (**Table 1**). The clinical significance of this mutation has remained uncertain. ACMG classification for the *LRP5* mutation presented it to be likely pathogenic (PS1, PM2, PP3). The *LGR4* gene mutation has not been entered in the dbSNP database, suggesting that it has never been reported. ACMG classification for the LGR4 mutations suggested it to be likely pathogenic (PM2, PM6, PP3). The majority of the Varsome prediction tools showed the LGR4 mutation to be damaging/deleterious; however, about half of the prediction tools suggested the LRP5 mutation to be damaging (**Table 2**).

**Table 1:** Sequencing depth and allele frequencies of LRP5 and LGR4 mutations.

Gene	Mutation	Zygosity	Read depth	Population allele frequency				
				1000 Genome_overall	1000 Genome_South Asians	gnomAD_overall	gnomAD_South Asians	IndiGenomes
LRP5	c.1199 C>T (p.Ala400Val)	Heterozygous	142	0.0003	0.002	0.0002	0.0007	0.001
LGR4	c.1403 A>C (p.Tyr468Ser)	Heterozygous	31	–	–	–	–	–

**Table 2:** In-silico predictions for the observed mutations.

Predictor tools	LRP5 scores	Indicative prediction	LGR4 scores	Indicative prediction
AlphaMissense	0.3068	Likely Benign	0.9185	Likely pathogenic
CADD (Phred score)	24	Deleterious	28.1	Deleterious
DANN	0.9993	Likely pathogenic	0.9919	Likely pathogenic
DEOGEN2	0.8309	Damaging	0.6395	Damaging
EIGEN	0.2404	Likely Benign	0.6531	Uncertain
EIGEN PC	0.2779	Likely Benign	0.6924	Uncertain
FATHMM	-2.67	Damaging	4.44	Tolerated
FATHMM-MKL	0.9866	Damaging	0.9956	Damaging
FATHMM-XF	0.9013	Damaging	0.9504	Damaging
LIST-S2	0.8843	Damaging	0.9445	Damaging
LRT	0.000302	Unknown	0	Deleterious
M-CAP	0.6834	Damaging	0.05395	Damaging
Mutation assessor	2.185	Uncertain	3.195	Medium
MutationTaster	1	Disease causing	1	Disease causing
MutPred2	0.609	• Altered transmembrane protein- • Altered ordered interface	0.850711	• Altered transmembrane protein- • Gain of disulphide linkage at C471 • Loss of sulfation at Y468
MVP	0.5754	Uncertain	0.691	Likely pathogenic
PrimateAI	0.5537	Tolerated	0.7157	Tolerated
PROVEAN	-3.14	Damaging	-7.9	Damaging
SIFT	0.051	Tolerated	0	Damaging
SIFT4G	0.062	Tolerated	0.001	Damaging
BayesDel addAF	-0.1061	Tolerated	0.2823	Damaging
BayesDel noAF	-0.0721	Tolerated	0.1677	Damaging
MetaLR	0.72	Damaging	0.1163	Tolerated
MetaRNN	0.8012	Damaging	0.8827	Damaging
MetaSVM	0.5647	Damaging	-1.0375	Tolerated
REVEL	0.598	–	0.522	Uncertain

**Figure 2:**
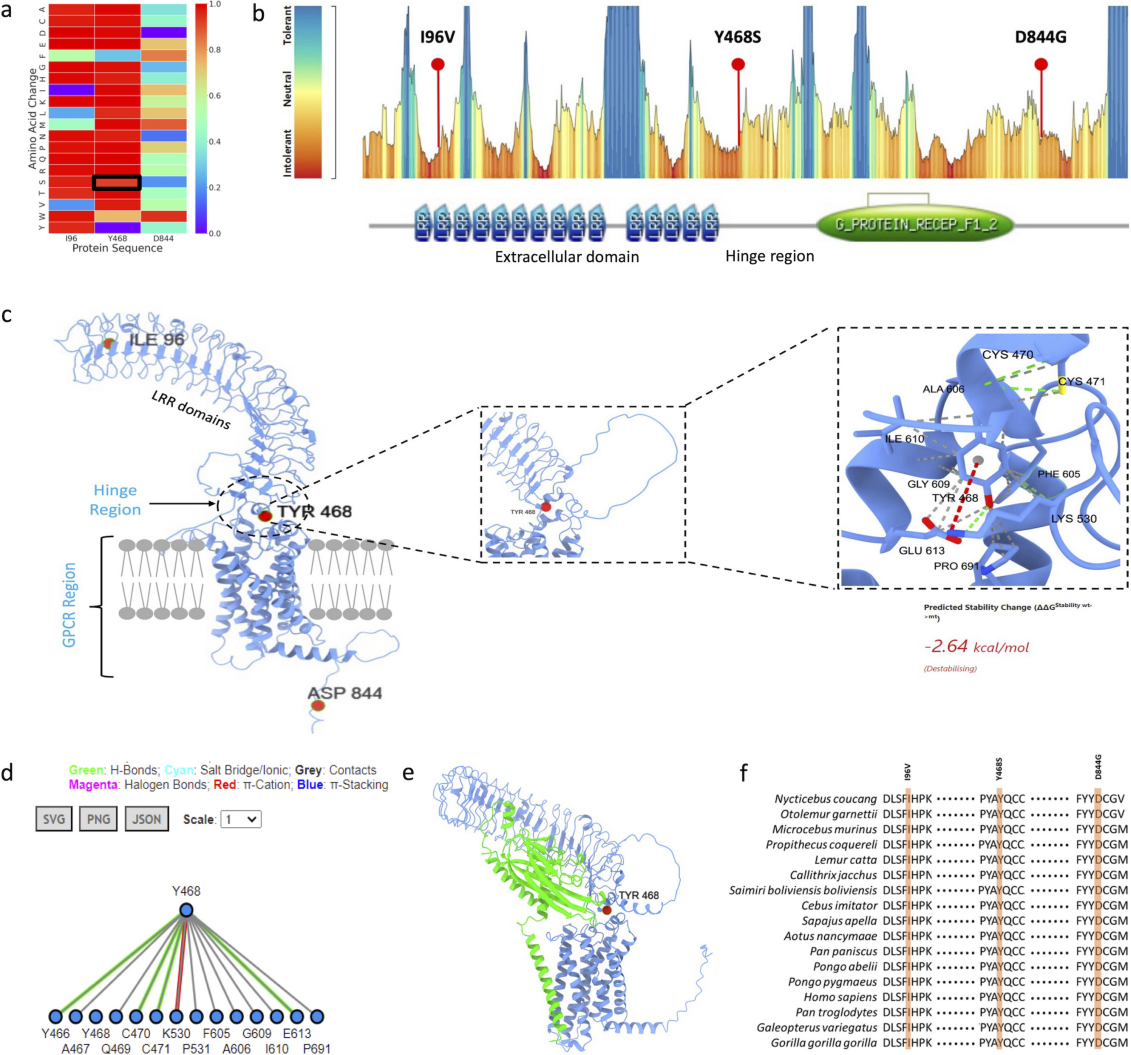
**(A)** AlphaMissense prediction score presented as a heatmap, highlighting p.Tyr468Ser substitution as pathogenic, **(B)** MetaDome prediction indicating p.Tyr468Ser substitution in an intolerant region, located within the hinge region of the protein, **(C)** Structural model of LGR4 displaying all pathogenic mutations, with specific emphasis on the intramolecular interactions involving the Tyr468 residue, **(D)** 2D interaction fingerprint diagram showcasing the detailed molecular interactions of the Tyr468 residue, **(E)** Modelled structure of LGR4 bound to RANKL, demonstrating the interface and interaction, **(F)** Evolutionary conservation analysis of the identified pathogenic variants along with the Tyr468 residue, highlighting its significance.

AlphaMissense (11) and MetaDome (12) tools indicated high pathogenicity of LGR4 mutation (**Figures 2A, B**). Tyr468 is positioned within the hinge region of the LGR4 protein, and interacts with a number of amino acids, which probably is meant to flexibility to this region of the protein (**Figures 2C, D**). RANKL binds with LGR4 in a way involving the Tyr468 position (**Figure 2E**), which further supports its functional significance. Tyr468 position is evolutionarily conserved across various mammalian species (**Figure 2F**). Structural analysis of LGR4 reveals that the side chain of the Tyr468 residue plays a critical role in maintaining the protein’s integrity. It participates in essential hydrogen bonding, cation-π interactions, and hydrophobic contacts with neighboring residues within the protein. Substitution of Tyr468 with serine (or with any other amino acid) is likely to disrupt these interactions, potentially leading to significant destabilization of the LGR4 protein structure and its functional properties. This observation is reinforced by the calculated ΔΔG value of -2.64 kcal/mol.

**Figure 3:**
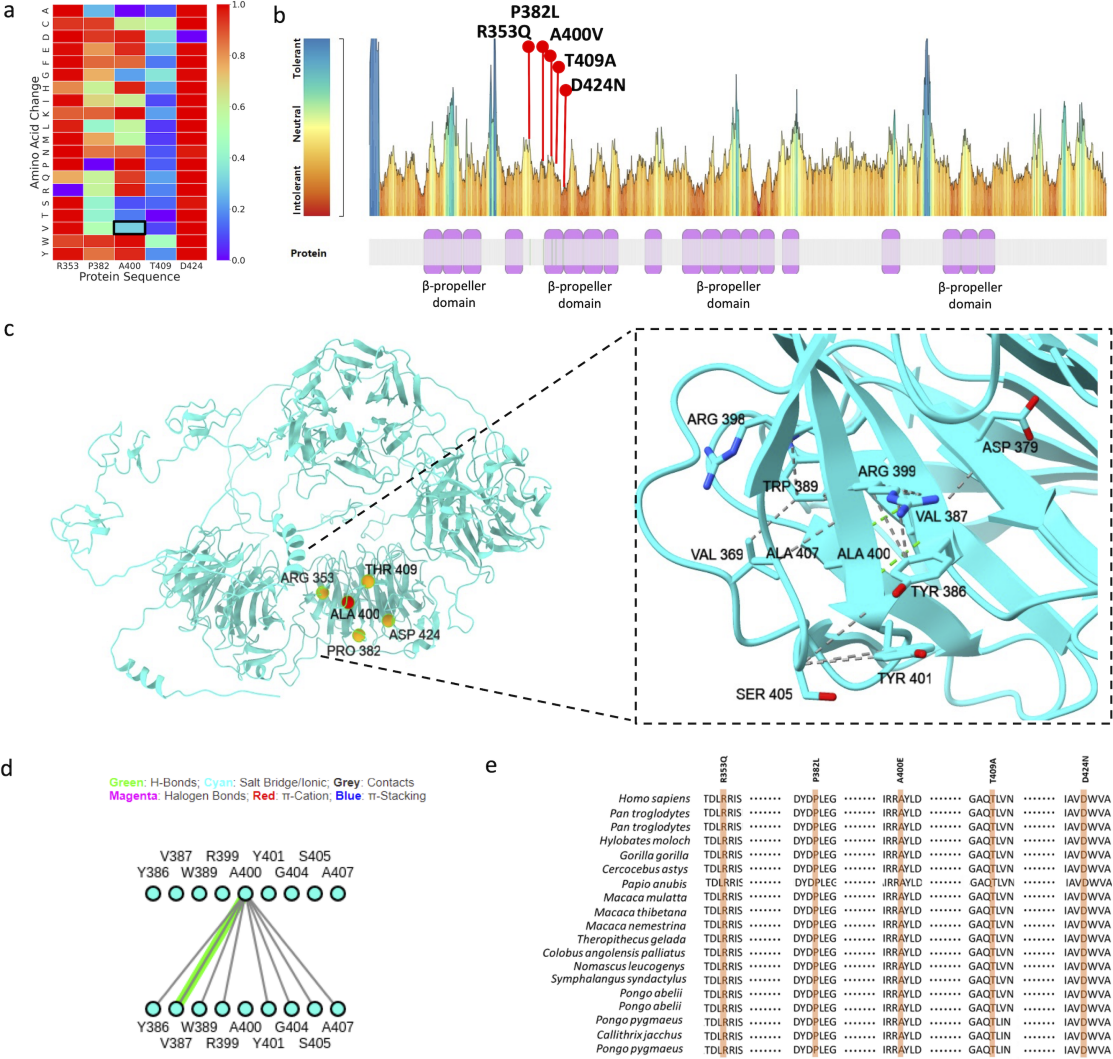
**(A)** AlphaMissense prediction score displayed as a heatmap, showing p. Ala400Val as a tolerated mutation, **(B)** MetaDome prediction showing p. Ala400Val substitution in mildly intolerant region, **(C)** Structural model of LRP5 illustrating all pathogenic mutations, with particular focus on the intramolecular interactions involving the Ala400 residue, **(D)** 2D interaction fingerprint diagram illustrating the molecular interactions of the Ala400 residue, **(E)** Evolutionary conservation analysis of the identified pathogenic variants along with the Ala400 residue, highlighting its significance.

AlphaMissense suggested low pathogenic potential for the LRP5 mutation (**Figure 3A**), but MetaDome suggested it to be potentially pathogenic (**Figure 3B**). A400 lies in the second β-propeller domain (**Figure 3B**), which is critical to Wnt binding and functions of the LRP5 receptor. Interestingly, other mutations in the second β-propeller domain are pathogenic (**Figure 3C**), and A400 interacts with several critical amino acids in this domain (**Figure 3D**), suggesting its functional significance. Further, the A400 residue is evolutionary conserved across mammalian species, highlighting its functional importance (**Figure 3E**).

**Figure 4:**
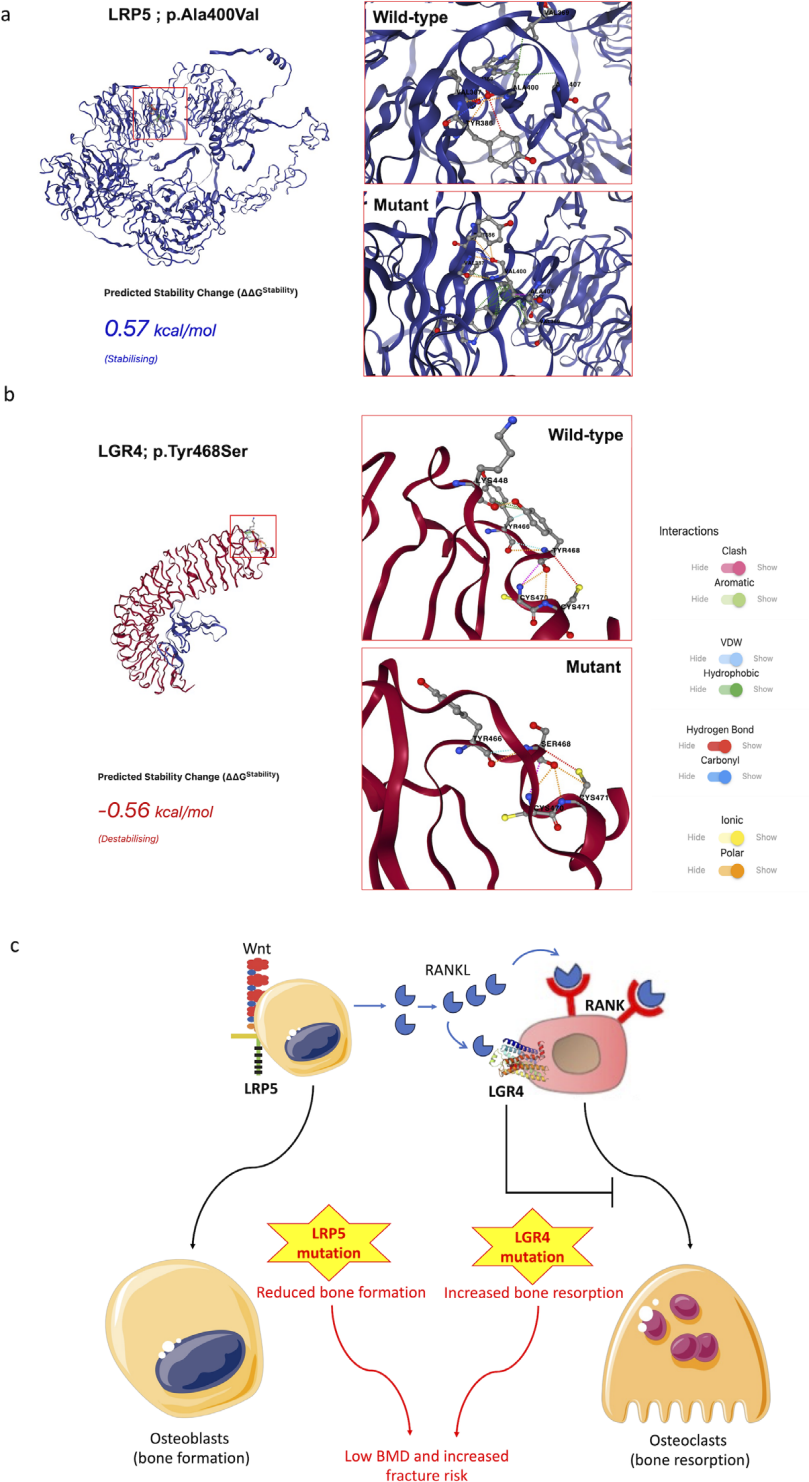
DynaMut2 analysis of the effects of mutations on LRP5 **(A)** and LGR4 **(B)** proteins. The interactions of the target amino acid residues in the wild type and mutant LGR4 and LRP5 proteins are shown. The LGR4 prediction used the PDB crystal structure file for this protein. Proposed mechanism of BMD loss in the present case **(C)**.

The mutation prediction tool MUpro showed unstable protein structures, suggesting scores of ‘-0.466’ and ‘-1.2969’ for LRP5 and LGR4, respectively. On the other hand, DynaMut2 showed the LRP5 mutation to be stabilizing and LGR4 mutation to be destabilizing (**Figures 4A, B**). The complete X-ray structure for LGR4 was not available; therefore, we also used AlphaFold model of LGR4 and observed a higher destabilizing effect of the LGR4 mutation on the complete structure (ΔΔG= -3.15Kcal/mol using DynaMut2 and -2.64Kcal/mol using DDMut).

## 4 Discussion

The studied patient experienced low BMD, severe anemia, scalp hair loss, weight loss, and progressive bone deformities leading to knee buckling and leg bending, accompanied by declining health from the age of 35 until her death at 61 years. Though she presented to the clinic for the first time with skeletal issues, it is apparent from her health records that the underlying mutation(s) affected the functioning of multiple organs. Genetic analysis revealed heterozygous mutations in the *LRP5* and *LGR4* genes that could explain the phenotype in this case. LRP5 is very well known to affect BMD and fracture risk (17). The gain-of-function mutations in the *LRP5* gene have been linked with increased BMD (17, 18), and the loss-of-function mutations have been linked with low BMD (19, 20). Mutations in the *LRP5* gene are known to cause osteoporosis-pseudoglioma (OPPG) syndrome in an autosomal recessive fashion. Heterozygous carriers for the LRP5 mutation display reduced BMD and osteoporosis (21, 22) and population-based studies suggest that common LRP5 variants contribute to general variations in BMD (23–25). A number of heterozygous, homozygous, and compound heterozygous mutations, including at the position of the present mutation (400^th^ amino acid), have been reported in OPPG patients with extraskeletal manifestations. Some of these mutations were functionally validated to result in the loss of signal transduction, suggesting their physiological impact (26). LRP5 mutations are in particular known to cause primary osteoporosis without the typical features of OI (8).

RANK/RANKL signaling is known to promote osteoclastogenesis, promoting bone resorption (27). LGR4 acts as a receptor for RANKL and thus competes with RANK for RANKL to negatively regulate osteoclast differentiation and bone resorption (28). This way, LGR4 activity keeps bone resorption in check. Accordingly, human mutations in the *LGR4* gene have been reported to cause low BMD and increased fracture risk (29, 30). A study on 1300 Chinese subjects from 390 nuclear families identified *LGR4* genetic polymorphisms to affect peak BMD (30). BMD has been seen as a quantitative trait from a genetic point of view. However, a study aimed at identifying genetic mutations resulting in pathologically low BMD identified a mutation in the *LGR4* gene that was strongly associated with low BMD and osteoporotic fractures (29). Thus, mutations alone in the *LGR4* gene can significantly affect BMD. Investigations over the last decade have found LGR4 to play a central role in the regulation of several physiological functions, including bone mineral density, and endocrine and metabolic diseases (31). LGR4 deletion also increases susceptibility to inflammatory bowel disease (32), which is an independent risk factor for BMD loss.

We believe that heterozygous mutations in the *LRP5* and *LGR4* genes contributed to a major loss of bone density in the present case. LRP5 promotes osteoblastogenesis, and LGR4 mitigates osteoclastogenesis. Mutation in the *LRP5* gene would reduce osteoblastogenesis, and mutation in the LGR4 would promote osteoclastogenesis, both contributing to BMD loss (**Figure 4C**). Both LRP5 and LGR4 have a wide distribution with significant expression across the liver, kidney, smooth muscles, female reproductive tract, salivary glands, adipose tissue, pancreas, brain, thyroid gland, and other organs (33). Their expressions in the liver are particularly high in comparison to most other organs, suggesting their significant role in liver functions. LRP5 regulates the canonical Wnt signaling across a number of these organs, suggesting its multitude of physiological effects (34), which may explain extraskeletal features due to LRP5 mutants, including in the present case. LRP5 mutations have been previously reported to result in hepatic cystogenesis due to the loss of Wnt signaling (35). We believe that A400V in the *LRP5* gene must have contributed to the overall low BMD and liver failure in this individual. Investigations over the last decade have found LGR4 to play a central role in the regulation of several physiological functions, including bone mineral density, and endocrine and metabolic diseases (31). LGR4 is also known to protect hepatocytes from injury. LGR4 is expressed in mature hepatocytes and protects them from TNF-α-induced cell death (36). In the absence of LGR4, the liver becomes particularly susceptible to acute injury. LGR4 has also been shown to be important for liver regeneration (37). The association of LGR4 with liver health suggests the contribution of the LGR4 mutation to liver failure in this patient. LGR4 has also been shown to be important for hematopoiesis (38), the deficiency of which may explain severe anemia in this patient.

The *LRP5* gene has significant expression in the female reproductive tract, which might explain early menopause in this case. Similarly, LGR4 has also been found to be important for ovarian development and gonadogenesis (39). A study investigating the genetic variants associated with premature ovarian insufficiency undertook whole exome sequencing and identified 195 pathogenic/likely pathogenic variants in a number of genes, including LGR4 (40). This, along with other physiological abnormalities due to the multi-organ role of LGR4, may explain early menopause in this patient. Osteomalacia, cold sensation in the feet, and knuckle pigmentation in the patent could be due to vitamin D deficiency. In conclusion, we report two likely pathogenic mutations in the *LRP5* and *LGR4* genes in a patient with osteoporosis, recurrent fractures, and poor overall health who died at the age of 61 years due to acute liver failure. The selected mutations appear to explain most of the phenotypic features and physiological abnormalities observed in this patient. A detailed description of the patient’s phenotype and health status with whole exome sequencing and confirmation of mutations by Sanger sequencing are strengths of this study. The lack of functional assays to support the pathogenic nature of these compound mutations is a limitation of our study. Further research on these genes in animal models and human patients would add strength to our findings.

## Data Availability

All data about this manuscript are available from the corresponding author on a reasonable request.
